# Predictive Factors of Spontaneous Reporting of Adverse Drug Reactions among Community Pharmacists

**DOI:** 10.1371/journal.pone.0155517

**Published:** 2016-05-18

**Authors:** Yun Mi Yu, Euni Lee, Bon Sun Koo, Kyeong Hye Jeong, Kyung Hee Choi, Lee Kyung Kang, Mo Se Lee, Kwang Hoon Choi, Jung Mi Oh, Wan Gyoon Shin

**Affiliations:** 1 College of Pharmacy & Research Institute of Pharmaceutical Sciences, Seoul National University, Seoul, South Korea; 2 College of Pharmacy, Chung-Ang University, Seoul, South Korea; 3 College of Pharmacy, Suncheon University, Suncheon, Joellanam-do, South Korea; 4 Regional Pharmacovigilance Center, Korean Pharmaceutical Association, Seoul, South Korea; Royal College of Surgeons, IRELAND

## Abstract

**Purpose:**

To evaluate the association between spontaneous reporting (SR) and the knowledge, attitude, and needs of community pharmacists (CPs), using a questionnaire following a conceptual model known as the mixed model of knowledge-attitude-practices and the satisfaction of needs.

**Methods:**

**S**elf-administered questionnaires were used with a nationwide convenience sample of CPs between September 1, 2014 and November 25, 2014 in Korea. The association between SR and the predictive factors was evaluated using multivariate logistic regression analysis.

**Results:**

In total, 1,001 questionnaires were analyzed. The mean age of the respondents and the number of years spent in community pharmacy practice were 45.6 years and 15.3 years, respectively. CPs with experience of SR was 29.4%. Being older than 60 (ORadj, 0.16; 95% CI, 0.06–0.42), having prior experience with adverse drug reactions (ADR) (ORadj, 6.46; 95% CI, 2.46–16.98), having higher specific knowledge of SR (ORadj, 3.58; 95% CI, 1.96–6.56), and having less concern about the obstacles to SR (ORadj, 0.36; 95% CI, 0.23–0.57) were significant contributing factors to SR. The main obstacles to SR included perception of ADRs as ‘not serious ADR’ (77.9%), ‘already well known ADR’ (81.5%), and ‘uncertain about causality’ (73.3%). CPs without reporting experience had greater concerns related to the reporting method and the liability of the pharmacy than those with reporting experience (p<0.05).

**Conclusions:**

Findings from our study showed around one in three CPs had ADR reporting experience in Korea, while 87.1% had prior experience with ADR cases. The knowledge of SR, prior experience of ADR, and less concern about the obstacles to SR were contributing factors for reporting levels.

## Introduction

When a new drug is reviewed on its efficacy and safety for its approval, all possible side effects of the drug cannot be anticipated based on preapproval studies because the studies include only several hundred to several thousand patients [1]. Therefore, the World Health Organization has set up the International Programme for Adverse Reaction Monitoring and played a leading role in global drug safety monitoring [2]. Other health authorities such as the European Medicines Agency (EMA) and the U.S. Food and Drug Administration (FDA) have worked collaboratively to improve pharmacovigilance as reflected by a recent report documenting an increased level of collaboration and sharing information between EMA and FDA to advance regulatory excellence in global community.[3]

The post-marketing surveillance is central to the monitoring of high priority adverse drug reaction (ADR) cases, and for gaining a perception of practical drug safety [4, 5]. Spontaneous ADR reporting is an essential responsibility of health care professionals as it safeguards patients from harm during drug post-marketing periods. Active surveillance and diligent ADR reporting by health care professionals lead the improvement in drug safety by the early detection of rare or delayed ADR and will ultimately contribute to the improvement of patient care [5, 6] In the U.S., pharmacists were recognized as one of the most important healthcare providers in spontaneous ADR reporting [7]. More specifically, in the Netherlands, Spain, and Portugal, community pharmacists (CPs) played an important role in spontaneous reporting (SR) [8, 9]. The ADR reporting rate by CPs in these countries ranged from 12.0% to 28.9% [8].

Limited data are available on the ADR report rates by Korean pharmacists. A report by the Korean Food and Drug Administration using 2008 data stated that “pharmacists” were the third ranking reporters (9.7%), led by doctors (69.0%) and consumers (17.6%) [10]. Inadequacy in their knowledge about the SR system was also described in a study involving 90 pharmacists [11]. However, the low ADR reporting rates by pharmacists were primarily based on SR through the Pharmacovigilance Research Network [12] before active involvement of the Korean Pharmaceutical Association (KPA), including CPs [13].

In spite of the establishment of the Pharmacovigilance Research Network [12] and the expansion of the Regional Pharmacovigilance Centers in Korea since 2009, not much is known about the ADR reporting rates by CPs; very few studies have highlighted the active roles of pharmacists in pharmacovigilance, and even fewer studies have specified the factors that enhance or hinder the reporting using validated survey tools driven by a conceptual framework. With the KPA being designated as one of the 22 Regional Pharmacovigilance Centers in Korea from January 2013 [13], an evaluation of the reporting behavior and factors influencing SR by CPs will provide valuable information which can be used to create educational intervention programs.

Therefore, this study aimed to evaluate the association between the SR behavior and the predictive factors among Korean pharmacists in a community pharmacy setting, using a questionnaire driven by the mixed theoretical model [14] of knowledge-attitude-practices [15] and the satisfaction of needs [16].

## Methods

### Study design

A cross-sectional survey study was conducted using a self-administered questionnaire with a nationwide convenience sample of CPs in Korea. The questionnaire was developed and then distributed via an online or paper-based method. Online recruitment was carried out on the website of PM2000, a community pharmacy’s billing program that is used by the majority of community pharmacies in Korea and periodic pop-up reminders were delivered on the PM2000 website. To solicit survey responses for pharmacists who did not prefer online platform or used a different billing program, paper-based survey was created and distributed to pharmacists at two national-level conferences. The data were collected by the participating pharmacists between September 1, 2014 and November 25, 2014.

All survey participants provided their written informed consent to participate in this study while ensuring confidentiality to meet our ethical standards. A full ethical review was made for all procedures following the protocol approved by the Institutional Review Board (IRB) of Seoul National University and the study was approved by the IRB (IRB No. E1410/001-011).

### Survey instrument

To construct the survey, a mixed theoretical model developed by Herdeiro and colleagues [14] was adopted in this study. The mixed model included the knowledge-attitude-practices model [15] and the theory of satisfaction of needs [16] that has been utilized in other studies on ADR reporting [17–19].

The survey instrument contained the extrinsic and the intrinsic factors [14]. The extrinsic factors included the pharmacy environment and the pharmacist’s relationship with patients, physicians, and public health administrators. The intrinsic factors were personal and professional variables, knowledge of SR, attitude to spontaneous ADR reporting, and reporting habits. Questions related to the pharmacy environment included the location of the pharmacy, the classification of nearby hospitals, and the daily prescription volume. The questions about the pharmacist’s relationship with patients, physicians, and public health administrators were related to obstacles to ADR reporting and strategies to improve SR.

The questions concerning personal and professional factors consisted of age, gender, length of career in community pharmacies, and prior experience of ADR. The questions for general knowledge of SR related to knowledge about the SR system, the designation of KPA as one of the Regional Pharmacovigilance Centers, legal responsibilities related to the reporting of serious events, and affiliation with KPA. The questions about eligible reporters, i.e., “Who can report?” and reportable items, i.e., “What to report?” regarding SR were included to gather information about specific knowledge of SR. The questions about attitude were related to the sense of ADR reporting being the professional duty of pharmacists, obstacles to ADR reporting, and strategies to improve SR. Data regarding CPs’ reporting history was obtained by asking about their experience of spontaneous ADR reporting.

The questions about obstacles to SR were based on Inman’s seven reasons [20] and published studies about attitudes to SR [17, 18, 21–25], and totaled eleven questions. These questions included the attitude to SR as part of the intrinsic factors and also addressed CPs’ relationships with patients, physicians, and public health administrators as part of the extrinsic factors. The obstacles, including CPs’ perceptions of ADR were ‘not serious ADR’, ‘already well-known ADR’, ‘uncertain about causality’, ‘unaware of the reporting method’, ‘complexity of reporting procedure’, ‘lack of time for reporting’, ‘liability of the pharmacy’, ‘lack of compensation’, ‘wouldn’t make a real improvement’, ‘doctor did not want’, and ‘patient did not want’.

Eight questions on strategies to improve SR were based on a qualitative study by Vallano and colleagues [26] and published literature on under-reporting [19, 21, 23, 27–29]. The questions included ‘provision of causality assessment to the reporter’, ‘continuous promotion and education on reporting’, ‘provision of educational resources based on the reports’, ‘provision of a manual on handling ADR inquires’, ‘simplification of reporting procedure’, ‘confidentiality of the reporter’s identity’, ‘legal protection of reporter’, and ‘appropriate compensation for reporting’.

The questions about obstacles to SR and strategies to improve reporting utilized a five-point Likert-type scale where five points were assigned for ‘strongly agree’, four points for ‘agree’, three points for ‘neutral’, two points for ‘disagree’, and one point for ‘strongly disagree’. For an analytical purpose, the data were converted to binary data by combining “strongly agree” and “agree” as an agreement and “neutral,” “disagree,” and “strongly disagree” as a neutral/disagreement.

The questionnaire was piloted with four investigators of this study, four CPs, and four pharmacy students and a few questions were rephrased to improve the clarity of the questionnaire.

### Sample size

Based on a small-scale in-house exploratory study indicating that 20% of CPs had ever reported ADR cases and an expected increase of the reporters after the designation of KPA as a regional pharmacovigilance center, a sample size of 740 subjects was calculated as adequate to detect a 10% expected frequency increase with 80% power and 5% α-error. (Epi Info™ 7.1.5, Centers for Prevention and Control, Atlanta, GA). An additional recruitment was made to consider a 20% withdrawal rate [30], constituting 10% of the subjects discontinuing their online survey in early phase and 10% of the subjects submitting insufficient data without key information, respectively.

### Statistical analysis

The internal consistency reliability and the construct validity of the instrument in the knowledge and attitude domains were assessed using Cronbach’s α coefficient and exploratory factor analysis, respectively. A Cronbach’s α ≥ 0.7 was considered adequate for internal consistency [31]. The cutoff value of 0.5 in the Kaiser-Meyer-Olkin measure of sampling adequacy and a p-value < 0.001 in Bartlett’s test of sphericity were used to ensure the fitness of the data set [32]. Eigenvalues > 1 as the reference and examination of the scree plot were used to determine the number of factors. Items that demonstrated a loading ≥ 0.4 were considered as the corresponding factors [33].

Descriptive statistics were used to summarize the characteristics of the study population. Mean and standard deviations were used to describe the central tendency for the continuous variables such as age and the length of career in community pharmacies, whereas frequencies and percentages were used for the categorical variables. Bivariate and multivariate associations between the independent variables (i.e., prior experience of ADR, knowledge of SR) and the outcome variable (experience of spontaneous ADR reporting) were evaluated using univariate and multivariate logistic regression analysis, respectively. The degree of association was described by odds ratios with their corresponding 95% confidence intervals. Data analysis was performed using SPSS version 21.0 (SPSS Inc., Chicago, IL) and the significance level was set at p < 0.05.

## Results

From September 1, 2014 to November 25, 2014, a total of 1,004 of the 1,315 invited CPs participated in this study from 7 major cities and 9 provinces representing all the administrative districts in Korea. The total number of questionnaires included in the final analysis was 1,001, i.e., 541 online and 460 from the paper-based survey; three questionnaires that did not include responses on the key questions about the experience of spontaneous ADR reporting were excluded.

The mean (± SD) age of participating CPs was 45.6 (± 10.9) years and males comprised 41.5% of the population (Table 1). The mean (± SD) years of their careers in community pharmacies was 15.3 (± 10.4). The daily prescription volume per pharmacy was less than 75 in 47.0% of all participating pharmacists and the majority of the pharmacists worked at pharmacies close to private clinics (82.9%).

**Table 1:** Population demographics (n = 1001).

Characteristics	Value
Age, mean ± SD (years)	45.6 ± 10.9
Sex, n (%)	
Male	415 (41.5)
Female	577 (57.6)
Unknown	9 (0.9)
Career in community pharmacy, mean ± SD (years)	15.3 ± 10.4
Affiliated with KPA, n (%)	288 (28.8)
Location of the pharmacy, n (%)	
Metropolitan area	617 (61.6)
Rural area	383 (38.3)
Unknown	1 (0.1)
Classification of nearby hospitals*, n (%)	
Private clinic	830 (82.9)
Hospital	111 (11.1)
General hospital	96 (9.6)
Superior general hospital	44 (4.4)
Unknown	6 (0.6)
Daily prescription volume per pharmacy, n (%)	
≤ 75	470 (47.0)
76–150	323 (32.3)
≥ 151	192 (19.2)
Unknown	16 (1.6)
Experience of spontaneous ADR reporting, n (%)	294 (29.4)

*Classification of medical institutions by the number of inpatient beds in Korea: private clinic–less than 30 beds, hospital–30 to 99 beds, general hospital–100 to 299 beds, superior general hospital–more than 299 beds.

Abbreviations: KPA, Korean Pharmaceutical Association; ADR, adverse drug reaction.

While 87.1% of the CPs had prior experience with ADR in our study population, only 29.4% of all participating CPs stated their experience with spontaneous ADR reporting. About three quarters (77.0%) of CPs knew about the existence of the SR system and 95.5% of the CPs acknowledged SR as a professional duty.

### Reliability and validity

In the internal consistency reliability analysis, Cronbach’s α for items related to the knowledge of SR and items relating to the attitude towards obstacles to SR was 0.58 and 0.67, respectively, which were below the acceptable value of 0.7. Cronbach’s α for items relating to the attitude towards ways of improving SR was 0.72, which indicated moderate internal consistency reliability.

In an analysis for construct validity, the Kaiser-Meyer-Olkin (p = 0.693) test and Bartlett’s test of sphericity (p < 0.001) were used, which showed that the items of knowledge, attitude towards obstacles, and attitude towards ways to improve SR were appropriate for exploratory factor analysis. Table 2 shows the factor loading for each item of knowledge and attitude towards SR. Factor analysis indicated that 4 items of SR knowledge loaded on a single factor. The item loadings ranged from 0.48 to 0.81. Factor analysis confirmed that the 11 items relating to attitude towards obstacles were three dimensional, explaining a total variance of 52.4%. The three dimensions, interpreted in the light of Inman’s seven reasons, were related to ignorance or diffidence, lethargy or methodological issues, and fear or indifference. In the attitude items on strategies to improve SR, two factors were extracted, describing a total variance of 51.1%. These two factors were the provision of education or educational resources and the simplification of the method and the institutional safeguards for reporters.

**Table 2:** Internal consistency reliability and factor loading on the items of knowledge and attitude towards spontaneous ADR reporting.

Item	Cronbach’s α if item deleted	Factor		
		1	2	3
**General knowledge of SR (n = 974)**				
Knowledge of SR system	0.45	0.77*		
Knowledge of RPC-KPA	0.38	0.81*		
Knowledge of related laws	0.56	0.57*		
Affiliated with KPA	0.60	0.49*		
Eigenvalue		1.81		
Cumulative variance explained (%)		45.21		
**Attitude towards obstacles to SR (n = 856)**				
Not serious ADR	0.67	-0.02	0.83*	-0.06
Already well-known ADR	0.67	-0.02	0.85*	-0.01
Uncertain about causality	0.66	0.14	0.55*	0.13
Unaware of the reporting method	0.65	-0.01	0.08	0.80*
Complexity of reporting procedure	0.62	0.18	0.11	0.80*
Lack of time for reporting	0.66	0.22	-0.09	0.53*
Liability of the pharmacy	0.64	0.60*	-0.01	0.28
Lack of compensation	0.64	0.59*	0.00	0.19
No real improvement	0.63	0.64*	0.10	0.13
Doctor did not want	0.63	0.79*	0.04	0.02
Patient did not want	0.65	0.68*	0.03	-0.02
Eigenvalue		2.75	1.71	1.30
Cumulative variance explained		21.01	36.84	52.36
**Attitude towards strategies to improve SR (n = 892)**				
Provision of causality assessment to the reporter	0.69	0.71*	0.11	
Continuous promotion and education on reporting	0.70	0.66*	0.11	
Provision of educational resources based on reports	0.68	0.68*	0.24	
Provision of a manual on handling ADR inquiries	0.68	0.72*	0.16	
Simplification of reporting procedure	0.67	0.33	0.59*	
Confidentiality of the reporter’s identity	0.74	0.06	0.84*	
Legal protection of reporter	0.68	0.14	0.81*	
Appropriate compensation for reporting	0.69	0.13	0.46*	
Eigenvalue		2.89	1.20	
Cumulative variance explained		25.85	51.14	

* Item loading > 0.4.

Abbreviations: ADR, adverse drug reaction; SR, spontaneous reporting; RPC-KPA, Regional Pharmacovigilance Center—Korean Pharmaceutical Association.

### Contributing factors for spontaneous reporting

The comparison of the personal and professional factors, knowledge, attitude, and pharmacy environments, and how these relate to experience of spontaneous ADR reporting, is presented in Table 3. Pharmacists older than 60 years were less likely to report ADR than pharmacists aged 40 and less (p<0.001). Having a prior experience of ADR was a significant predictor for ADR reporting (ORadj, 6.46; 95% CI, 2.46–16.98; p<0.001). For analytical purposes, the data on the knowledge of SR, the attitude towards obstacles to SR, and the attitude towards strategies to improve SR were divided into three groups in approximately equal proportions as ‘high’, ‘moderate’ and ‘low’. CPs with higher knowledge of SR were more likely to recognise who can report and what to report than were those with lower knowledge (p<0.05). The attitude towards obstacles to SR was the influencing factor of SR. The reporting group showed lower concern about the obstacles than the non-reporting group (p<0.05)

**Table 3:** Contributing factors for the spontaneous reporting of adverse drug reactions.

Characteristics	Reporting group, n (%)	Non- reporting group, n (%)	OR_unadj_ (95% CI)	OR_adj_ (95% CI)*
Age group				
≤ 40 years	108 (37.0)	226 (32.5)	1.0	1.0
41–50 years	105 (36.0)	243 (34.9)	0.90 (0.65–1.25)	0.91 (0.59–1.40)
51–60 years	64 (21.9)	151 (21.7)	0.88 (0.61–1.29)	0.71 (0.43–1.16)
≥ 61 years	15 (5.1)	76 (10.9)	0.41 (0.23–0.75)^†^	0.16 (0.06–0.42)^†^
Sex				
Female	142 (48.6)	435 (62.1)	1.0	1.0
Male	150 (51.4)	265 (37.9)	1.73 (1.32–2.29)^†^	1.39 (0.95–2.03)
Career in community pharmacy				
≤ 10 years	98 (34.1)	303 (44.6)	1.0	1.0
11–20 years	109 (38.0)	201 (29.6)	1.68 (1.21–2.32)^†^	1.62 (0.98–2.68)
≥ 21 years	80 (27.9)	175 (25.8)	1.41 (0.99–2.00)	1.79 (0.88–3.65)
Prior experience of ADR				
No	5 (1.7)	120 (17.1)	1.0	1.0
Yes	289 (98.3)	583 (82.9)	11.90 (4.81–29.43)^†^	6.46 (2.46–16.98)^†^
General knowledge of SR				
Low (0–1 item)	16 (5.5)	297 (43.4)	1.0	1.0
Moderate (2 items)	87 (30.1)	192 (28.0)	8.41 (4.79–14.77)^†^	11.71 (5.73–23.91)^†^
High (3–4 items)	186 (64.4)	196 (28.6)	17.62 (10.25–30.28)^†^	23.89 (11.80–48.35)^†^
Knowledge on eligible reporters and reportable items				
Low (0 item)	120 (40.8)	455 (64.7)	1.0	1.0
Moderate (1 item)	118 (40.1)	197 (28.0)	2.27 (1.68–3.08)^†^	1.56 (1.06–2.31)^†^
High (2 items)	56 (19.0)	51 (7.3)	4.16 (2.71–6.40)^†^	3.58 (1.96–6.56)^†^
Professional responsibility				
No	5 (1.7)	35 (5.0)	1.0	1.0
Yes	289 (98.3)	667 (95.0)	3.03 (1.18–7.82)^†^	2.29 (0.67–7.82)
Level of attitudes towards obstacles to SR				
Low (≤ 3 items)	132 (50.0)	166 (28.0)	1.0	1.0
Moderate (4–5 items)	81 (30.7)	198 (33.4)	0.51 (0.36–0.73)^†^	0.59 (0.38–0.91)^†^
High (≥ 6 items)	51 (19.3)	228 (38.5)	0.28 (0.19–0.41)^†^	0.36 (0.23–0.57)^†^
Attitudes towards strategies to improve SR				
Low (≤ 6 items)	80 (28.7)	158 (25.8)	1.0	1.0
Moderate (7 items)	66 (23.7)	146 (23.8)	0.89 (0.60–1.33)	0.84 (0.51–1.39)
High (8 items)	133 (47.7)	309 (50.4)	0.85 (0.60–1.19)	0.79 (0.51–1.24)
Location of pharmacy				
Rural areas	112 (38.2)	271 (38.3)	1.0	1.0
Metropolitan areas	181 (61.8)	436 (61.7)	1.00 (0.76–1.33)	0.96 (0.65–1.42)
Daily prescription volume				
≤ 75	138 (47.3)	332 (47.9)	1.0	1.0
76–150	94 (32.2)	229 (33.0)	0.99 (0.72–1.35)	0.85 (0.55–1.30)
≥151	60 (20.5)	132 (19.0)	1.09 (0.76–1.57)	1.10 (0.65–1.85)

*Multivariate analysis adjusting for all variables in the table based on 774 pharmacists.

^†^p < 0.05.

Abbreviations: OR_unadj_, unadjusted odds ratio; OR_adj_, adjusted odds ratio; CI, confidence interval; ADR, adverse drug reaction; SR, spontaneous reporting.

Gender, length of career in community pharmacies, and professional responsibility, were significantly associated with reporting in the crude analysis, but after adjustment of the remaining factors, the associations were not statistically significant. There was no difference in the attitude towards strategies to improve SR, and the pharmacy environment, such as the location of the pharmacy and the number of prescriptions, between the two groups. The prescription volume was not a significant predictor for SR.

### Attitude towards obstacles to spontaneous reporting

**Fig 1:**
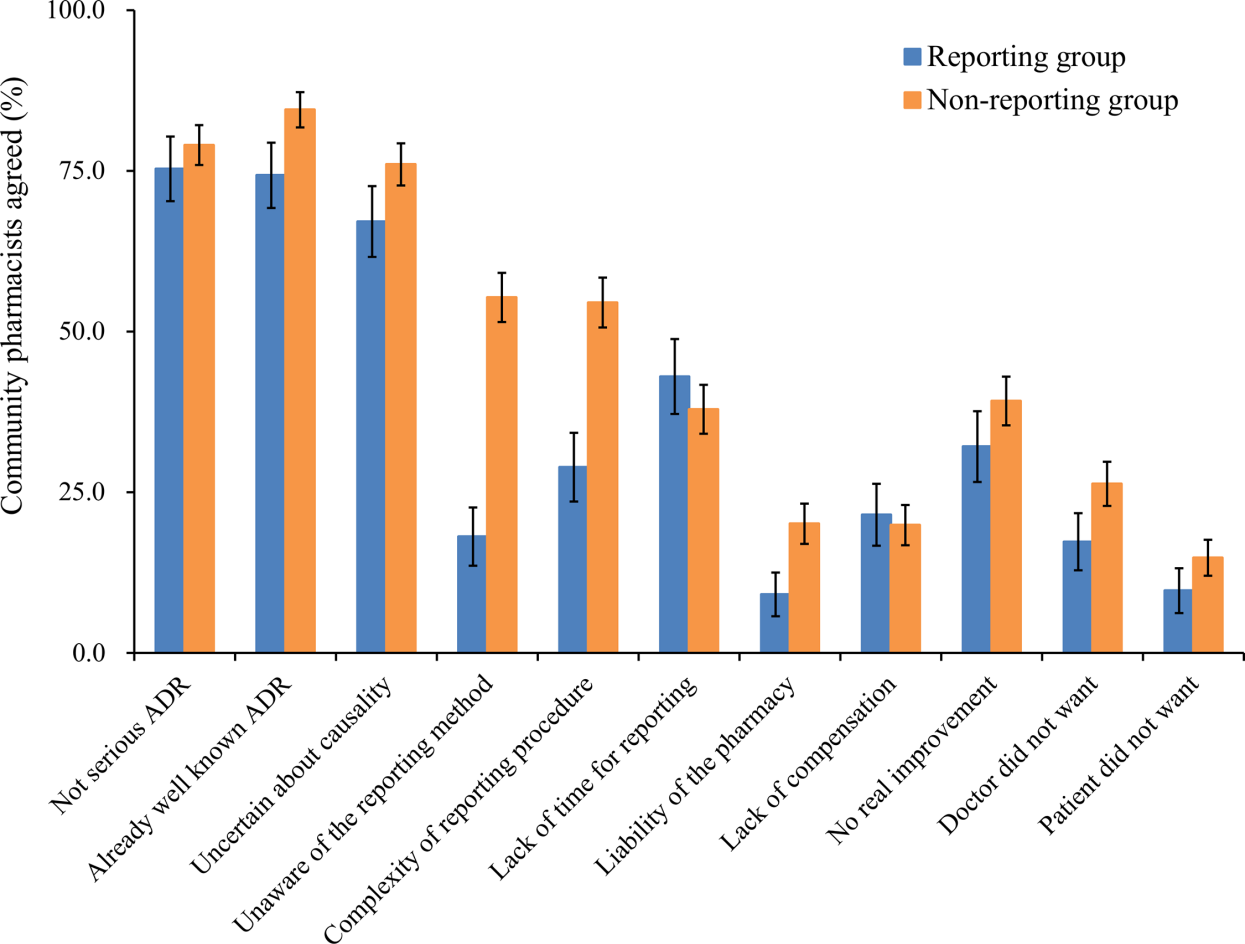
Attitude towards obstacles to adverse drug reaction reporting in community pharmacists. n = 898; reporting group (n = 277), non-reporting group (n = 621). Error bars represent 95% confidence interval of the percent agreement.

More than two-thirds of the population in both groups agreed that ‘not serious ADR’ (77.9%), ‘already well-known ADR’ (81.5%), and ‘uncertain about causality’ (73.3%) were the reasons for under-reporting (Fig 1). Except for ‘not serious ADR’, ‘lack of time for reporting’ and ‘lack of compensation’, the concerns about all the other types of obstacles were significantly higher in the non-reporting group (p<0.05). Particularly, the difference between the two groups was greater in ‘unaware of the reporting method’, ‘complexity of reporting procedure’ and ‘liability of the pharmacy’ than in the other items. ‘Unaware of the reporting method’ (ORunadj, 0.18; 95% CI, 0.13–0.25; p<0.001), ‘complex reporting procedure’ (ORunadj, 0.34; 95% CI, 0.25–0.46; p<0.001), and ‘liability of the pharmacy’ (ORunadj, 0.40; 95% CI, 0.25–0.62; p<0.001) were regarded as influential obstacles lowering the level of reporting.

### Attitude towards strategies to improve spontaneous reporting

More than 70% of the study population agreed on all eight strategic items for improving SR. Only two out of the eight items were significantly different between the reporting group and the non-reporting group. ‘Confidentiality of the reporter’s identity’ (ORunadj, 0.69; 95% CI, 0.50–0.95; p = 0.020) and ‘legal protection of reporter’ (ORunadj, 0.65; 95% CI, 0.44–0.97; p = 0.032) showed significantly lower rates of agreement in the reporting group (71.8%, 83.9%, respectively) compared to the non-reporting group (78.8%, 88.9%, respectively).

## Discussion

To the best of our knowledge, this is the first nationwide survey study of predictive factors of spontaneous ADR reporting in CPs in Korea. Dealing with not only the prescription drugs but also nonprescription drugs, nutritional supplements, and other pharmacy products, CPs are well-positioned to monitor and report on ADRs more extensively for outpatients [9]. However, very few studies have targeted CPs when evaluating the contributing factors for SR. It is also important to elicit the predictive factors of under-reporting, so that strategies to improve reporting by CPs can be projected from this study. An additional strength of our study is that the questionnaire was constructed on the basis of a conceptual framework, which helps to ensure that items both represent the practical realities and allow for extraction of explanatory factors associated with under-reporting [34, 35].

In this study, advanced age (≥ 61 years) was detected as a significant factor for ADR reporting; the elderly CPs were less likely to report ADRs than the young adult CPs. This result is consistent with a study on hospital pharmacists in China [27]. On the other hand, Irujo and colleagues showed a rise in the number of ADR reports with increasing age, but the age was not statistically significant in the multivariate-adjusted analysis [17]. The knowledge of SR showed a proportional correlation with the level of reporting in our study. A number of published studies, including a systematic review, also showed that the lack of knowledge of SR was a major cause of under-reporting [19, 25, 28, 36]. The tendency for under-reporting with old age and the lack of knowledge shown in our study implied the need for regular and ongoing continuing education (CE) regarding spontaneous ADR reporting. Several State Pharmacy Boards in the U.S. mandate CE with content on medication errors or patient safety related to SR. Such CE requirements can be an effective strategy to improve SR.

The main obstacles to SR were CPs’ perception of ADR as ‘not serious ADR’, ‘already well-known ADR’, and ‘uncertain about causality’ in both groups. This is consistent with other studies on CPs [17, 18] and hospital pharmacists [21, 27]. Although Su and colleagues highlighted the obstacles as inherent limitations for hospital pharmacists [27], published studies showed that this was recognized as a common challenge for physicians as well [19, 22, 24, 26, 37].

It is important to focus on another major obstacle with a higher proportion in the non-reporting group, which was the complexity of the reporting procedure. It would be necessary to develop a tool for easy access and to streamline the reporting process to the ADR reporting system in order to overcome this obstacle [23, 26, 28]. In this respect, the active involvement of the KPA in ADR reporting as a member of the Regional Pharmacovigilance Centers may be instrumental, as it has contributed to simplifying the reporting process by sharing the online interface with a major community pharmacy’s billing program [13].

Findings from our study indicated that more than 95% of the pharmacists reported SR as their professional duty, regardless of their previous reporting history. A number of studies corroborate with the findings from our study, embracing SR as a professional duty of pharmacists [18, 27, 28], while a systematic review on ADR under-reporting showed that one of the reasons for under-reporting was that pharmacists regard SR as being the physician’s duty rather than their own [25]. Therefore, strategies which inspire a sense of responsibility seem to be needed for improving spontaneous ADR reporting for CPs in Korea.

Considering the contributing factors and obstacles to SR in this study, one of the solutions for under-reporting can include simplifying the reporting process with automation in creating the report, providing feedback to and allowing an access to educational resources to the reporters, or supplying a manual on handling ADR inquiries. Finally, a strategy for preventing the under-reporting with aging can be continuous and persistent implementations of educational interventions, which would include information about the SR system, the purpose of SR, the method of reporting, and causality assessment. As the positive effects of educational interventions were documented in previous interventional research studies [23, 28, 38], practical training sessions built into the curricula of pharmacy colleges, or CE sessions for practicing pharmacists on medication safety, could be effective methods for improving SR activities [39].

This study has some limitations. First, we relied on the voluntary participation of convenience samples of CPs in this survey. This has the potential for selection bias with limited generalizability. If the participants with positive thoughts about SR are more involved, the results may be overestimated. Second, Cronbach’s α for items related to the knowledge of SR and items related to the attitude towards obstacles to SR was below the acceptable value of 0.7, while items related to the attitude towards strategies to improve SR was above the cutoff. The literature suggested possible reasons for lowering Cronbach’s α include a low number of items, levels with dichotomous responses, or multidimensionality [40, 41]. We believe our findings of α value less than 0.7 could be explained in part by the suggested reasons, which might reduce the internal consistency of our survey. In fact, we found a few studies with binary responses and multidimensionality used α value of 0.5 as their cutoff [42–44].

In conclusion, the findings from our study showed that less than one in three CPs had ADR reporting experience in Korea, while 87.1% had prior experience with ADR cases. The knowledge of SR, prior experience of ADR, and less concern about the obstacles to SR were contributing factors for reporting levels. Simplifying the reporting process, providing the feedback to the reporter, or continuously educating the senior CPs can be a potentially effective solution to increase reporting rates. Future studies are needed to create educational interventions and to evaluate its impact on improving SR among CPs in Korea.
